# Cholera in Tynemouth in 1831-2, 1848-9, and 1853

**Published:** 1855-12

**Authors:** E. Headlam Greenhow

**Affiliations:** Member of the Council of the Epidemiological Society, and Lecturer on Hygiene in the Grosvenor Place School of Medicine.


TRANSACTIONS

OF THE

EPIDEMIOLOGICAL SOCIETY OF LONDON.

---

### Papers and Communications.

---

### CHOLERA IN TYNEMOUTH IN 1831-2, 1848-9, AND 1853.

By E. Headlam Greenhow, M.D.,

Member of the Council of the Epidemiological Society, and Lecturer on Hygiene in the Grosvenor Place School of Medicine.

Late in the month of October 1831, cholera made its appearance at Sunderland, having, it was supposed, been introduced by a vessel from the infected port of Hamburgh. One morning towards the end of November, and about a month after the outbreak in Sunderland, the public of North Shields was much alarmed at learning that a suspicious case of illness had occurred in that town on the previous evening. The patient, an inmate of a common lodging-house, had been pronounced by the medical man who visited him to be suffering from cholera. Firmly impressed with the conviction that cholera was contagious the medical gentleman in attendance, to prevent the extension of the disease, secluded the inmates of the affected house by placing a constable at the door, where he remained all night. In this manner eight persons, besides the sick man, were confined, for a period of thirteen or fourteen hours, to the close, ill-ventilated atmosphere of a room not more than thirteen feet square by seven in height, happily, however, without any extension of the disease. On the following morning the patient was visited by all the medical practitioners of the town, including the writer of this account, at that time a medical student. The coldness and cramps which were stated to have existed on the previous evening had disappeared. The patient was warm, and the pulse, although feeble, was perfectly distinct; the only symptoms that remained which bore any resemblance to those ascribed to cholera being slight diarrhœa and almost incessant vomiting. The coldness, blueness of surface, absence of pulse and other symptoms which were supposed to be identified with the pestilence, being absent or having disappeared, it was almost unanimously agreed that the gentleman who first saw the case had been mistaken; and for the purpose of allaying the public excitement a placard to that effect, signed by all the leading medical practitioners, was profusely distributed throughout the neighbourhood; the constable was forthwith removed from the door, and the unhappy prisoners were re-admitted to free intercourse with the outside world. Although the man recovered from the immediate attack he passed into a typhoid condition, closely resembling what has since been termed the consecutive fever of cholera, and died at the expiration of ten days. At the *post mortem* examination, which was carefully conducted, no important organic lesion was detected, to account for death; the viscera were all tolerably healthy, and the gall bladder, as has since been so frequently observed in the bodies of persons who have died of cholera, was distended with bile. Few persons acquainted with that disease will now hesitate to acknowledge that the opinion of the medical gentleman who saw the patient on the evening of the attack was correct, and that this was really the first case of Asiatic cholera in North Shields.

Several days elapsed without the occurrence of any other cases of a suspicious nature; but on the 10th of December two were reported in different and distant parts of the town. Of these, one, a mendicant named McGuire, residing in a common lodging-house situated in a dirty court called Clayton's-yard, had come from Sunderland the day previous to the attack, bringing with him a considerable quantity of filthy rags; the other, who had neither left the town nor had had communication with any supposed source of contagion, was a widow in delicate health, living alone, and at least half a mile from the other patient. Both persons were said to be of intemperate habits. The woman died after a few hours' illness; the man recovered. The next case, which occurred on the 13th of December and proved fatal on the following day, was the wife of Dennis McGuire, the mendicant already named. On the 19th, three cases occurred in a district at least a mile from any of the former ones, and each of the three at a distance from each other, all of which were fatal on the day of attack. It was said that two of these had been in contact with sources of contagion. The most careful inquiry could elicit no communication with any infected source in the third. On the 20th, another case occurred which, like the last, could not be attributed to communication with a suspected source ; and on the same day a pauper nurse, of drunken habits, who had been sent from the workhouse to wait on Dennis McGuire and his wife, was likewise seized with the pestilence and died after a few hours' illness. From this time scattered cases continued to arise in various parts of the town, some of which were supposed to have received the contagion from their acquaintances, or, as it was more frequently stated, during a visit paid to one of the adjoining towns in which cholera was prevalent; whilst others, like some of those already named, were acknowledged by the keenest contagionists to have baffled all inquiries as to their origin.

Before proceeding to the further consideration of this, the first epidemic of cholera in North Shields, and in order to render what is to follow intelligible to persons unacquainted with the locality, a brief description of the district seems necessary. The parish of Tynemouth, of which North Shields forms the urban portion, is situated at the confluence of the Tyne with the sea, and constitutes the south-eastern angle of Northumberland. On the south it is washed by the river Tyne, and on the east by the German Ocean. The greater portion of the parish is elevated from fifty or sixty to between two and three hundred feet above the tidal datum; the only low lying land being a narrow ledge, upon which stands the original town of North Shields. This ledge, which is scarcely elevated above high water level, extends for about a mile along the river side, and rarely exceeds forty or fifty yards in breadth. From this the cliff rises abruptly to a height of from seventy to ninety feet, from the summit of which the land rises by a very gradual ascent towards the north and west. The original town of North Shields having been constructed at a time when traffic was ordinarily carried on by means of pack horses, consists of a line of long narrow streets, in most parts of which two vehicles cannot pass. From either side of these streets open lanes and courts which terminate on the one hand at the margin of the river, and on the other at the steep cliff up the face of which at frequent intervals are narrow and precipitous flights of stairs, leading to the upper town, and bordered on either side with houses. Less than a century ago, the wealthy shipowners, tradesmen, and other opulent inhabitants, resided entirely in this district, now called the Low Town; and even so recently as 1831, many tradesmen and other persons of respectable station continued to occupy houses attached to their places of business in the main street or the adjoining courts. In the meantime, many tolerably spacious streets and squares had been erected on the higher ground; and the High Town, which had thus sprung into existence, probably contained in 1831 about two-thirds of the entire population of the place, a proportion which is still maintained. From the nature and aspect of the ground, the upper town of North Shields, or Tynemouth as it is now called, is admirably placed for health, and affords great facilities for drainage, an advantage which has hitherto been almost entirely neglected. In some of the streets inhabited by the poorer classes, these natural advantages have in a great degree been neutralised by the unsystematic manner in which the land has been plotted out for building purposes, and by the partial adoption of the system, already described as common in the Low Town, of building courts and lanes which communicate by narrow and often covered passages with the main street, and for the most part possess the additional disadvantage of having no exit. The western extremity of the town is formed by a district called Milburn Place, consisting of several parallel streets, the land to the north and west of which is partly under cultivation, and partially occupied by long irregular rows of cottages, built as dwellings for the pit-men who work in the adjoining collieries. These pit-rows, as they are locally named, are frequently intersected by ditches, the ordinary receptacles for the slops and other refuse of the surrounding population; the roads are neither paved nor macadamized, and the cottages themselves are destitute of every kind of convenience, and frequently surrounded by pig styes. On the east, three-quarters of a mile from the outskirts of the town, lies the village of Tynemouth, pleasantly situated on a dry and elevated site adjoining the coast; between it and the town lies a deep dene, in the flat bottom of which are many manufactories, and which both in lowness of elevation and in the character of the houses and their occupants, very closely resembles the Low Town. Tynemouth proper (for the entire parish and borough takes its name from the village) consisted in 1831 of one very broad and spacious main street, parallel with which are two narrow ones communicating with it and each other by smaller cross streets. There was at that time no drainage to the village, and with the exception of a narrow track in the centre of the principal street, little attention was paid to the state of the roads, into which ashes, night soil and garbage of every description were indiscriminately thrown.

About two miles north of Tynemouth lies the fishing village of Cullercoats, also situated on the sea coast, at a good elevation, and possessing many advantages of situation, including a pure and most salubrious atmosphere. It is irregularly laid out, and consists of one broad street, with two or three narrow lanes and bye-courts opening out of it. Both here and at Tynemouth, with the exception of the better class of houses occupied by lodging-house keepers, and used during the summer and autumn by the numerous visitors from Newcastle and the adjoining counties, there is an almost total absence of water-closets and necessaries; hence in 1831 every vacant corner was employed as a place of deposit for ashes, and animal and vegetable refuse of all kinds, an evil which continues at Cullercoats at the present date. The like deficiency of these essential conveniences exists in many parts of North Shields, and in Chirton, an ancient village lying on the north-west outskirts of the town.

A careful inquiry into the amount of the privy accommodation in the town was made in the year 1849, when it was found that 2,838 families, consisting of 10,605 persons, had for their use only 983 privies. In Chirton and the rows of pit cottages on the western outskirts of the town, such conveniences scarcely existed; and one small district, the Ropery Banks, occupied by 100 families, was entirely destitute. Milburn Place, containing 487 families, had 104 privies, which, however, affords by no means an adequate idea of its real condition in this respect, for whilst every house in what is termed the front street which was formerly the residence of persons of a superior grade, possessed one of these conveniences, the houses designed for the poorer classes were almost without them. In the course of a very careful inquiry, instituted by the Town Council in 1850, it was found that when the dwellings of the poor were furnished with conveniences, these were either erected against the main wall of a house, thus allowing the more liquid portion of the contents to percolate through into the subsoil below the apartments, or, as was more common, within some shed or other place on the groundfloor, over which were rooms occupied as sleeping apartments. The chief exception to this was afforded by the North Street, in the rear of which, at a distance not exceeding ten or twelve yards from the back of the houses, was an open ditch, overhung by rude conveniences for the use of the adjoining houses, and which also received the drainage from a number of filthy pigstyes. The side of the Low Town which abuts on the river was found to be almost without privy accommodation. The entire district, which in 1849 contained 7,000 inhabitants, comprised in 1,666 families, had for their use only 62 of these conveniences, most of them appertaining to houses still occupied by respectable tradesmen.

The principal manufactories are chain cable and anchor works, gas works, a pottery, steam flour mills and engine factories, none of which are likely to prove injurious to health, excepting from the volumes of smoke cast into the air from their wide-mouthed chimneys. Such, however, is the excellency of the situation, and so constant the currents of air which exist in this narrow part of the island, that clouds of smoke are very rapidly dissipated, and do not long hang suspended over the place. Although the atmosphere is thus comparatively but little vitiated by local manufactories, it is much influenced by the numerous chemical works on the south side of the river, which incessantly pour into it enormous volumes of muriatic acid vapour, besides sulphurous acid fumes which escape during the process of manufacture. The extent of this acid impregnation of the atmosphere is well illustrated by its effect upon vegetable and insect life. Trees and shrubs which thirty years ago flourished in the centre of the town will exist there no longer, and swallows and many other summer birds that formerly abounded in great profusion, the insect food upon which they existed being destroyed, have almost entirely disappeared. The reality of the effect upon vegetable life has been frequently recognised by juries, who have awarded heavy damages from the owners of alkali works to the proprietors of pleasure grounds and gardens for the injury produced by these acid exhalations.

The town of North Shields is supplied with water from several sources. There are public water-works, consisting of three separate sets of reservoirs, fed from different sources, and so situated as to render contamination impossible, excepting so far as deleterious matters may be absorbed from the atmosphere, or the water may be vitiated by the exuviæ of the numerous weeds, insects, and other animal beings, which abound in the uncovered reservoirs. The water varies much in quality; one portion, derived mainly from land drainage, scarcely exceeds six degrees of hardness by Clarke's test, whilst another, collected in a large limestone quarry, is of fourteen degrees of hardness. Dr. Richardson, an eminent analytical chemist, of Newcastle-upon-Tyne, who was employed to make an analysis for the purpose of the public inquiry into the sanitary condition of Tynemouth, states, that there was no reason to believe the water to be impregnated with organic matter, arising from the infiltration of a town drainage; and, indeed, such contamination is simply impossible, the reservoirs being at a distance from, and much elevated above, the level of all existing sewers.

A very small proportion of the houses derive their water supply directly from the mains, only three hundred out of about four thousand dwellings being thus supplied in 1849. Most of the inhabitants rely upon stand-pipes, locally called pants, at which the water is retailed, and upon perambulating dealers, who sell a very fair wholesome water obtained from ponds and springs at a little distance north of the town, and which are likewise uncontaminated by drainage. Many private residences of the better class possess wells of tolerbly good water, which vary much in quality, and often possess chalybeate properties.

The population of the entire parish, which amounted to 21,899 in 1831, had increased in 1841 to 27,249, and at the last census to 30,524. A rough but tolerably accurate estimate of its distribution, would probably assign one-fourth of the whole at each of these periods as inhabitants of the outlying villages and rural district; of the remainder, about one-third would be apportioned to the township of North Shields, already distinguished as the Low Town, and the remainder to the High Town.

As I have, unfortunately, been unable to obtain correct meteorological returns for the periods of the prevalence of cholera, I may here state from recollection, that the winter of 1831-32 was singularly open and mild, the only snow that fell being towards the end of January, when there were a few days of slight frost. The autumn of 1849 was warm and close; the atmosphere was singularly still during the existence of cholera, the prevailing wind being from the east, which is unusual at that season; the weather was bright and dry; twice or thrice there were thunder storms of some severity, which invariably for a brief space mitigated the pestilence.

The outbreak of cholera, the commencement of which, late in the year 1831, has already been detailed, lasted until the 25th of March, 1832. During the fifteen weeks of its continuance, 258 cases were reported to the Board of Health for Tynemouth nominated by the Privy Council, of which a careful examination of the mortuary register in the parish church shows that 139 proved fatal. This information is perfectly trustworthy, there being at that time no extra-parochial burial place: and, in consequence of the over-crowded state of the ordinary burial ground, a piece of unconsecrated ground, adjoining the churchyard, was set aside by the authorities for the interment of such persons as might die of cholera. A note of the cause of death was also, in every instance, added to the register in the church books, an exact transcript of which has been obtained to assist in the preparation of this paper. From the 25th of March to the 15th of July, 1832, there was an entire cessation of cholera in the parish of Tynemouth, no case of the disease being reported to the Board of Health, nor any deaths registered in the church books. On the latter day a fresh outbreak of the epidemic took place, which, continuing until the 6th of November following, carried off 125 victims. The number of cases is uncertain, the returns to the Board of Health appearing to have been less regularly made than on the former occasion, only 169 cases being recorded in the register kept by the secretary, the present mayor of Tynemouth, who has obligingly allowed me the use of that document for the present purpose. There is also an entire omission of the particulars of the cases, usually given by the medical men in each of their earlier reports. The second outbreak appears to have commenced simultaneously in several different localities, and not in one place from which it might be supposed to have extended as from a focus. There is therefore no reason to believe that it originated in importation. Indeed, such has never been asserted to have been the case: and if it were so, the only documentary evidence now procurable is directly opposed to such an opinion.

Dividing the town into districts, it is found that the disease on both these occasions was principally confined to a few localities. Although some of the worst of those attacked in the spring were re-visited by the pestilence in the autumnal outbreak, yet in the main at its second appearance cholera selected situations for its visit, which had hitherto either altogether or very nearly escaped. In the village of Tynemouth, wherein only one case occurred during the earlier months of the year, twenty-one took place in the autumn, of which thirteen proved fatal; the resident population probably not exceeding eight hundred. At that time, and indeed until within the last three years, the inhabitants of the village were entirely dependent on wells for their supply of water. The whole of the poorer classes, and many of the richer, derived this necessary of life from a very deep and never-failing well, the water of which is uncontaminated by drainage and perfectly free from organic impurities. Like almost all the well water in Tynemouth, it is brackish and unwholesome, as is well shewn by the fact that horses when first taken there speedily get out of condition, as well as by the frequency of attacks of English cholera and diarrhœa of unusual obstinacy in the summer and autumn of ordinary seasons. Cullercoats, which entirely escaped the earlier epidemic, was visited by cholera in the autumn, at which season the atmosphere is more than ordinarily vitiated by the effluvia from decaying organic remains. Out of a population which can scarcely have exceeded five hundred, eleven fell victims to the pestilence, being a greater amount of mortality from a single disease in the short space of a few weeks in this usually healthy village, than the average annual mortality of the entire county of Northumberland. Milburne Place and the township of North Shields were severely visited in both epidemics, sixty-four deaths having occurred in these districts during the spring visitation, and sixty-two in the autumn. Chirton also suffered severely: there were fifty-eight deaths in the two visitations, the larger portion being in the spring, and forty-one of the whole amongst the mining population, whose cottages, although built in an open situation, are often placed back to back, thus preventing free ventilation; often have pigstyes and dunghills in their immediate neighbourhood, and possess no privy or convenience of any kind. The miners themselves constitute a peculiar race, easily distinguished by dress and features, and are usually remarkable for the contrast between their cleanly personal habits and the neat and comfortable aspect of the interior of their dwellings, and their filthy and unwholesome state out of doors. Nine deaths occurred at the Low Lights, the district already described as lying in the dene between North Shields and Tynemouth. The total mortality from cholera in the Upper Town only amounted to twenty-five, sixteen of these being within a short distance of Clayton's Yard, in which the first recognised case of the pestilence took place on the 10th December 1831.

It is thus sufficiently apparent, that the cholera at its first appearance in Tynemouth selected the dirtiest situations for its seat. In fact, it prevailed almost exclusively in those parts of the town the atmosphere of which was most vitiated by the exhalations given off by the various kinds of organic matter, which the absence of proper necessaries and ash-pits caused to be accumulated either within the houses or in close proximity thereto.

From the 6th of November 1832 until the autumn of 1848, Tynemouth was free from cholera as an epidemic. Nevertheless, scarcely a season passed in which one or more sporadic cases, closely resembling the pestilential disease both in symptoms and the rapidity and fatal termination of their course, did not occur. During the ten years between 1837 and 1848 I saw four or five cases which, had they occurred during an epidemic visitation, would undoubtedly have been called Asiatic or pestilential cholera.

Early in the autumn of 1848, Dr. Dempster, assistant-surgeon, 33rd regiment, sent for the writer of this paper to visit a private soldier who was suffering from the symptoms of "approaching collapse"; the evacuations were of the rice-water character; the surface was cool, and the pulse exceedingly languid This man recovered. Shortly afterwards a still more severe case came under my notice in private practice, which also terminated favourably. Between the month of October and the end of the year several deaths were attributed to cholera; all of these, however, were either imported cases or persons who, although residing within the Tynemouth registration district which is conterminous with the union, were not inhabitants of the parish. The first really indigenous cases were in the month of March 1849, and took place in Pumpwell Lane, a small, dirty, undrained court near the west end of the town, the frequent seat of fever and other epidemic or endemic diseases. Three out of four persons attacked by the disease in the same house died. One of the rooms on the ground floor of the house in which these persons resided was used as a place of deposit for ashes, night soil and other impure garbage. The house itself being situated at the foot of a gradually sloping bank on-to the surface of which the slops of the houses above were thrown, the other ground-floor room, oceupied by the victims of cholera, was continually kept damp by the percolation of the liquid from above through the old and ruinous wall; occasionally this was so abundant that the occupants were in the habit of digging a shallow pool by the fire-side for its reception. From the month of March until that of July there was no death from cholera. The first cases that occurred at the latter period merit a detailed account, because, by many persons, they were believed to afford distinct proof of the contagious nature of cholera; with what justice will be presently apparent. The particulars of the supposed introduction of cholera into Tynemouth by contagion, in accordance with the statement made at the time, are briefly narrated at page 306 of the *Report on Epidemic Cholera*, published by the College of Physicians. It is there stated, that the disease was imported from Durham by a man who had there buried his wife and daughter, dead of the disease. On his return home, it is said, he took the complaint himself, and died of the consecutive fever; the woman who nursed him also took the disease, and, returning to her own house, situated in a different part of the town, there died, and from thence, as a focus, the epidemic gradually extended itself over the district. It is quite true that such was, at the time, the generally-accepted version of the introduction of cholera into North Shields in the summer of 1849. At the close of the epidemic in October, a more careful inquiry was instituted into the circumstances. The following account, obtained from the son of the man who was reported to have been the means of introducing the disease, contains the real facts, and, taken in conjunction with the preceding statement, affords a good illustration of the slender evidence upon which, very often, the reputed importation of the pestilence depends.

"Mrs. H. went to Durham, Sunday, June 10th, 1849. Mr. H. went to see her, Saturday, June 16th, and returned to Shields on Monday, June 18th. Up to the 20th Mrs. H. had no symptoms of cholera, but on Wednesday the 20th she took poorly, and purging commenced on the afternoon of the same day. She died the following morning, June 21st. Mr. H. took the bowel complaint on Wednesday, June 27th, and died on July 2nd, 1849."

It should be observed that Mr. H. did not return to Durham after June 18th, so that any infection he received must have been communicated previous to the illness of his wife, from whom he was supposed to have received it. Indeed, it was particularly named by her son that his mother, who had gone to Durham in consequence of ill health, had expressed herself as being very decidedly better on the Sunday and Monday of her husband's visit. The evidence in favour of the importation of the pestilence is thus defective in the most essential point. It has never been stated that Mr. H. received the infection from any one but his wife, and, if from her, is is evident he must have done so two days before she herself manifested the slightest appearance of illness. That the woman who nursed Mr. H. died of cholera proves nothing: she was of intemperate habits, and having passed three nights and days in the same apartment with the sick man, which was on the ground floor, she was doubtless exposed to the same atmosphere and local causes, if such were in existence. It is also erroneous to say that the disease extended from her house as from a focus, for there is not one tittle of evidence to prove that any subsequent case had been in communication with her, and the presumptive evidence is opposed to it, for the next cases were in distant parts of the town, and among persons by no means likely to have had communication either directly or indirectly with the supposed centre of contagion.

The next case that occurred was on the 8th of July, on which day a lady of advanced age residing alone, and who had been particularly prone to diarrhœa, was seized with symptoms of cholera and died on the day of attack. There was not the slightest ground for believing in the existence of contagion in this case, for the patient had not left the house for several weeks previous to her death. On the 10th of July, two days after the last case, a seaman but recently returned from sea in good health and free from diarrhœa, was taken with cholera, and, recovering from the stage of collapse, died of consecutive fever on the 22nd. In the meantime his mother, who nursed him, was likewise taken with the same disease, which hurried her off, after a brief illness, on July 17th. In a house on a line with that occupied by the last two patients, and within twenty or thirty yards of it, two deaths, those of a father and his daughter, occurred on the 27th and 29th of July. No communication is said to have taken place between the last two sets of cases, indeed, the contrary was asserted at the time. At the same time, it is only fair to admit that such intercourse may have occurred; an admission which is nevertheless unimportant, since the man first seized was never believed to have received the disease from infection. On a careful examination of the houses, street and court in which the last four cases occurred, no causes of insalubrity could be detected; they were accordingly considered as examples of the capriciousness which has been ascribed to this pestilence, which, it is said, occasionally avoiding unhealthy localities, selects its victims from places where the sanitary arrangements are good, and nothing, either in the locality or the habits and health of the sufferers, would lead us to infer their peculiar liability. About two years afterwards, in the course of an inquiry into the sanitary state of the borough and especially of the drainage, it was discovered that an ancient, long-neglected, and foul drain, passed either immediately underneath the floor or close to the outside wall of the room in which Mr. H. resided, and the atmosphere of which the nurse, who also died, inhaled for three days and nights. Passing onwards, underneath a house of a better description, in which there was, it is said, no case of cholera, the drain passed below the rooms occupied by the father and daughter who died on the 27th and 29th of July, and close by that in which the seaman and his mother resided, whence it found its way into a common sewer. Whether this drain had any part in the localisation of the cholera in these houses I pretend not to say, as no evidence has been afforded that any sensible effluvia arose from it. It is at the same time notorious that such nuisances are often least noticed by those most exposed to their influence. At any rate the circumstance appeared sufficiently interesting to deserve the space here afforded to it. None of the latter cases were in the neighbourhood of the court in which Mr. H.'s nurse resided, and from which it is supposed that the epidemic radiated. Indeed no other case occurred in that portion of the town until eighteen days after her death, when there was a solitary one at the distance of more than half a mile from her residence.

The next series of cases possesses a double interest, since they also are said to have arisen from importation, and many of the victims were Jews, who have frequently, but as it would appear erroneously, been stated to enjoy a comparative immunity from this pestilence. On the 29th or 30th of July, a Jew pedlar, residing in Reed-street, and not more than seventy or eighty yards distant from Clayton's-yard, in which several of the earlier cases occurred in 1831, was taken with cholera. This man, like Mr. H. already named, was said to have introduced the disease from one of the adjoining towns. There is proof, however, that two cases had previously occurred in the same street, one of them in the very next house to that in which the Jew resided. The medical gentleman who attended them, states that he could discover no evidence of infection in the first of the two, but infers that the second, who acted as nurse to the former, and was seized two days afterwards, had received the disease from her patient. In the same house in which the Jew resided six other persons, all of them Jews, likewise had the pestilence, which also shewed itself in Clayton's-yard, Bird's-yard, Beacon-street, and Charlotte-street, places within a hundred yards of the house in which the Jew lived. The whole of these streets and courts were at that time undrained, filthy and inhabited by persons of a very poor class; many of the houses were dirty and overcrowded, and the excretæ and other refuse matters of the population were either deposited in small ash-pits in close proximity with the houses, or thrown into the ruinous and half-stagnant channels at the sides of the main streets, which are themselves so constructed as to be but indifferently ventilated. Four days after the outbreak in Reed-street, two cases of confirmed cholera appeared amongst a family of Prussian Jews, residing in Bland-square, a close ill-ventilated court in the Low town. It was said at the time that these persons received the disease through intercourse with the Reed-street cases. Such intercourse had undoubtedly existed, but on a careful inquiry made at the time, it was ascertained that neither of the persons attacked had left the court. If the disease in these cases originated in contagion, the virus must therefore have been conveyed to them by some member of the family who did not himself suffer from it. Upon this point there is no documentary evidence, but the investigation made at the time conveyed the impression that there was no good reason for believing in the contagious origin of these cases. This opinion is very much strengthened by the circumstance that two other fatal cases of cholera happened in the same court during the epidemic, and that almost every person in the place had diarrhœa of greater or less severity. From this period the disease became general, and any further attempt to trace the cases to a contagious source, or to show the impossibility of its having thus originated in any individual instances, would be nugatory.

The sanitary state of North Shields, including both the High and Low Town, had retrograded since the cholera visitation of 1831, whilst the population had considerably increased. Many houses in the Low Town, occupied at the time of the former visitation by persons of a superior class, were now let out in single rooms as dwellings for the humbler classes. The same change of occupants had taken place in several streets and rows of cottages in other parts of the district which, formerly the residence of industrious mechanics, ship captains, and persons in comfortable circumstances, had passed into the occupation of tramps, low Irish, and other persons of an inferior grade and of dirty habits. At no period was the sanitary state of North Shields so bad as for a few years previous to, and at the period of, its visitation by cholera in 1849. Notwithstanding the most praiseworthy and diligent endeavours of the Board of Guardians to cleanse the town and remove nuisances during the epidemic, a special sanitary committee, aided by an ample staff of officers, including two medical inspectors, being temporarily appointed for the purpose, an inquiry, conducted by the writer of this paper in conjunction with a committee of the Town Council in January 1850, shewed the town fully to merit the character here assigned to it. In the report of the committee, which was based upon facts noted at the moment of inspection, and cordially adopted both by the members of the committee and by the Town Council, it is stated that the town was very partially and imperfectly drained; that drains were in many instances carried underneath houses, so as to prove a great source of annoyance and disease to their inhabitants; and that the construction of branch drains was so faulty, that the foul and loathsome air of the sewers rises into and pollutes the atmosphere of dwellings. As to scavenging and street cleansing, the best and most public streets were reported as far from clean, and the lanes, bye-streets, and more retired situations, as deplorably dirty. Heaps of mud and refuse frequently remained for many days at the corners of the streets, and all manner of animal and vegetable refuse and dirty water were said to be thrown into the open gutters and untrapped gully holes. In the lanes, courts, and back alleys, large accumulations of filth were abundantly met with in immediate proximity to dwelling houses, even empty rooms being sometimes used in lieu of midden-steads by the inhabitants of the courts and stairs of the Low Town. Large dunghills were reported to exist in the middle of some of the most populous courts and bye-streets of the borough, the contents of which were often allowed to accumulate until the quantity became large enough to vitiate the surrounding atmosphere for a considerable distance. There was such a dunghill in Clayton's Yard where the earliest acknowledged case of cholera took place in 1831; and a still larger one, from which sixty loads of fermenting manure were removed a day or two after the visit of the Committee, was situated in the Causeway bank, very near to the house in which the unfortunate patient and his eight fellow-prisoners were confined for so many hours in November 1831. Open and stagnant ditches, ill-constructed drains, and ruinous channels for surface drainage, which allowed the foul water to stand until evaporated under the influence of the sun and wind, wells used as cesspools, pig-styes, and leaky middens, the drainage from which often ran into the street, were reported as nuisances from which no part of the borough was perfectly exempt. Amongst other causes of insalubrity, that arising from the practice of slaughtering cattle in the butchers' shops, was especially named. One consequence of this custom was, that the offal and refuse were commonly thrown into an open dunghill in some neighbouring court or yard, where it was allowed to remain and putrify in the immediate vicinage of dwellings, to the great annoyance of the occupants and injury of the public health. An apt illustration of the magnitude of this evil and of the danger of interference with such accumulations during an epidemic period, was afforded by a court, in which was a dunghill of this kind in a filthy and overflowing state. On the occurrence of a case of cholera in the court, the authorities immediately, with well-intentioned but injudicious zeal, ordered it to be cleansed without the employment of disinfectants. The result of this rash act at a period of pestilential epidemic was the occurrence of several additional cases, and of a general outburst of diarrhœa among the inhabitants of the court, which was situated in a part of the town otherwise quite free from the disease. One very pertinent instance of the influence exercised by untrapped gullies in localising the cholera, fell under my own observation. In front of two houses of a better class, situated in a broad and airy street, and in an elevated and open situation, was one of the dirty channels for surface drainage to which reference has already been made; immediately in front of the house, which I shall call No. 2, was an untrapped gully, near which the drains from the sinks of both houses, after passing underneath them, joined the main sewer. Early in the morning of one day towards the end of July, a severe case of diarrhœa occurred in No. 1. At the time of my visit the smell from the gully, both at the door and in the house itself, was so offensive as to induce me to advise the removal of the entire family at once into the country, enforcing my recommendation with the assertion, that if cholera, as was at that time anticipated, should prevail to any extent in the neighbourhood, the occupants of these houses would scarcely escape. The advice was adopted, the patient speedily recovered and the rest of the family escaped. A few weeks afterwards, two deaths from the pestilence rapidly took place in No. 2, and although the family were removed on the very day after the occurrence of the first death, every individual member of it was subsequently ill with diarrhœa.

Whilst the sanitary condition of North Shields had thus retrograded during the seventeen years that elapsed between the first visitation of cholera in the early winter of 1831-2 and its return in 1848-9, the village of Tynemouth had been materially improved. Many houses of a superior class had in the meantime been erected, and the entire character of the place altered for the better; drains were made in the principal streets; the channels, in which water had formerly remained stagnant for months together, were reconstructed; the gullies were trapped, and many nuisances, formerly prevalent, were prevented by the activity of the police; the streets were macadamised, levelled, and put into a much better state of repair, and the channels and gullies were occasionally cleansed. Probably as a result of this improvement in its sanitary state, Tynemouth sustained no mortality from cholera throughout the second epidemic. Four cases, none of which terminated fatally, did indeed occur. Two of them were imported. A third was that of a blacksmith, whose workshop, situated at the verge of a steep cliff overhanging the sea, was immediately above the outfall of one of the sewers and close to an offensive open privy common to the inhabitants of several houses. The fourth case was that of a child, the whole of whose family had diarrhœa most severely; the house in which they lived had no back windows or other means for thorough ventilation, and against the main wall were placed the privies of two houses behind, the smell from which was at all times very evident when the doors and windows were closed. Moreover, the house itself was situated in a dirty and narrow part of one of the smaller streets, and in a locality in which fever of bad type had prevailed on two or three occasions within a few preceding years.

Referring again to the question of contagion, it may not be out of place to observe that the blacksmith lived in a part of the village where there was but little diarrhœa, although this milder form of the epidemic and dysentery were generally prevalent at the time when cholera was devastating North Shields. In the same room where the man himself lay prostrate on his bed, and for several days almost unable to move, his wife and children lived, slept, and ate. Notwithstanding my most urgent remonstrances, two or three of the latter were often crawling over the sick man's bed, and could scarcely fail to come in immediate contact, both with the excretions and with the breath and other exhalations from his body; yet they all escaped the pestilence: the wife alone, sometime after her husband was convalescent, being attacked with bowel complaint in so mild a form that she was able to continue her ordinary avocations.

Whilst cholera was prevailing in North Shields, and diarrhœa and dysentery among the inhabitants of Tynemouth, the troops quartered in Tynemouth Castle enjoyed the most perfect immunity. Their number was about two hundred men and officers, besides women and children. Probably the entire population within the castle walls, including civilians, was about three hundred. As soon as cholera appeared in North Shields the condition of the barracks was carefully investigated, all sources of atmospheric impurity being removed. The drains and privies were frequently flushed, and quick-lime and chloride of lime profusely employed as disinfectants; fires were occasionably lighted in all the barrack-rooms, the floors of which were cleansed by dry scrubbing; and to prevent the accumulation of slops and refuse, an inspection of the barrack-rooms, stairs, and offices, was made at frequent but uncertain intervals by the medical man in charge. Attention was, likewise, bestowed upon the provisions, the meat being baked instead of boiled; and all unnecessary exertion on the part of the men was avoided. In accordance with instructions received from the Director-general of the Army Medical department, the men were confined to barracks as soon as cholera appeared in the neighbourhood. As this was found to be both very irksome and depressing, the gates were opened two days later and the men allowed to go into the village and surrounding country, but prohibited from visiting the tainted district of North Shields; the more effectually to prevent which, non-commissioned officers were stationed at the entrance of the two roads leading thither from the village. Although every case of indisposition, however trivial, was at once admitted into hospital,* not more than half-a-dozen cases of mild diarrhœa and one of dysentery took place within the barrack-

* These statements are based upon a full knowledge of the circumstances, for the author was in medical charge of the troops and military hospital at the period referred to.

walls during the entire course of the epidemic, these complaints being, as already said, most rife in the adjoining village during the whole period of the epidemic visitation in North Shields.

Almost *pari passu* with the occurrence of the first cases in Reed-street and of the later cases in Norfolk-street, the epidemic appeared in its former haunts in the township of Chirton. Cases occurred at the end of July and early in August amongst the pitmen's cottages at Percy Main, a district in which there were 41 deaths in 1831. But little improvement had been effected in the interim, although the population had much diminished from the closure of two adjoining collieries. In the same locality upwards of eleven fatal cases occurred in 1849. Here, as elsewhere, the cases were speedily so numerous and generally distributed over the tainted district, as to baffle all attempts accurately to trace the course of the epidemic in reference to its propagation by contagion. The medical gentleman who reported the two earliest cases was the same who stated that the Jew, Barnard Israel, had received the disease from infection; it is, therefore, not unfair to presume that had he received any evidence that the Chirton cases arose from that cause it would have been stated in his report.

According to the Registrar-general's report on cholera, the Tynemouth registration district, which contained in 1851 a population of 64,230 persons, sustained a mortality of 815 from cholera and 89 from diarrhœa. Of these about half, or 411 deaths from cholera and 30 from diarrhœa, were in the outlying, rural and mining districts, and in the small seaport town of Blyth included in the union, but beyond the limits of the parish. The mortality within the parish of Tynemouth during the same period amounted to 404 from cholera and 59 from diarrhœa. This mortality, like that of 1831-2, was very unequally distributed: for instance, the township of North Shields, with a probable population of 8,500, had, between July 2nd and November 13th, 1849, 426 cases and 203 deaths, whilst the township of Tynemouth, with a population of nearly 14,000, had only 176 cases and 95 deaths. A minuter investigation shews that even these numbers, widely as they differ, fail to convey an accurate impression of the manner in which the pestilence selected the places of its visit. Thus, in the four weeks that intervened between August 20th and Sept. 18th, there were 23 deaths from cholera in Dotwick-street, the residence of 79 families comprising 349 persons, being, in round numbers, at the rate of six in the hundred. At one extremity of Dotwick-street is Pumpwell Lane, where the earliest deaths occurred, and at the other lies Milburn-place, in which, as we shall presently see, the mortality was very considerable. In an open and more elevated district, immediately behind Dotwick-street, the exact population of which has not been ascertained, but which cannot have exceeded 150, there were 11 deaths in the same period. Milburn-place contained, at the time of the visitation, 1,500 persons. The mortality in five weeks was upwards of 60, but it is worthy of note that the fewest deaths, in proportion, took place in South-street, every house in which has a yard and privy, whilst it was largest in North-street and on the north side of the street, behind which existed the before-named foul and open ditch. Here, although the street is from 60 to 70 feet above high-water mark and from 35 to 40 above Dotwick-street, the mortality was almost as great as in the latter, there having been 30 deaths out of 577 persons. The largest mortality of all was in Pumpwell-lane, where, including the three deaths in March, 8 out of 46 persons succumbed to the pestilence. Taking the entire district of Milburn-place, Dotwick-street, Mount Pleasant, and such of the immediately adjacent cottages as are comprised within the township of North Shields, and which in all contained rather under 2,300 inhabitants, the deaths exceeded 100; the total mortality in the subregistration district, of which this is a portion, being 243 out of a gross population of above 12,000. The next most fatal district in North Shields was Duke-street, which, with a population of 560, sustained a mortality of 12, most of which occurred in the bye courts which are particularly confined, dirty, and ill-ventilated. Several of the cases were in one notorious lodging-house for tramps; but in the absence of more precise information, I forbear the attempt to allocate minutely either the cases or deaths. An idea may, however, be formed of the condition of the people, when it is said, that for the accommodation of 196 families, there were only six privies, all of them private ones, thus leaving 191 families utterly unprovided with a convenience so essential to health, comfort, and decency.

Before passing to the consideration of the epidemic in the High Town, one circumstance seems worthy of mention. A new and very superior dry dock, of considerable size, was being constructed at the time of the visitation. At that period the workmen were occupied in concreting the bottom previous to flooring it; out of the men thus employed whilst none escaped diarrhœa many had cholera, of which disease four or five died in different and distant parts of the town.

Cullercoats, like Tynemouth, had been somewhat improved in the interval since 1832. Although the streets were still used for the reception of offal and other waste substances of an organic nature, they were regularly cleansed from such impurities every morning. Out of a population of between 700 and 800, sixteen had cholera, of whom five or six died.

Of the ninety-five deaths in the Tynemouth sub-registration district, fourteen were in Reed-street, and including these, twenty-eight were within a circuit of two hundred yards round Clayton's-yard, of which so much has already been said respecting the outbreak in 1831. In Norfolk-street, where some of the earliest cases occurred in 1849, the disease lingered until the conclusion of the epidemic, nine out of fourteen cases proving fatal. In Bolton's Yard, where the large dunghill was imprudently emptied during the epidemic, three persons died. Within a small circle round the house in which one of the two fatal cases occurred on Dec. 10th, 1831, there were seventeen deaths in 1849, including in that number the two that ostensibly arose from an untrapped gully. In the Low Lights and its immediate neighbourhood, nine deaths took place; and in Stephenson-street, a long, dirty street, without lateral communications with the adjoining streets, and from either side of which courts without exit branch off, eleven cases out of eighteen proved fatal. Thus, eighteen deaths only are left for the entire remainder of the High Town, a very small proportion indeed, if compared with the mortality from cholera, in the localities of which special notice has been taken.

Not only, however, did cholera manifest a liking for particular localities; in these localities it affected particular houses. Time would fail me did I attempt to go fully into this part of its history; and, in fact, reliable evidence on this head is not now to be obtained. A careful examination of the records in existence shews that more than one case of collapse occurred in fifty houses; more than one death in upwards of forty; and that in several nearly all the inmates were swept away. In one house in North-street every individual died, and the house was closed for long afterwards. Out of seven Jews, inhabitants of the same house in Reed-street, five died: the other two also were ill, but recovered. Four members of one family, in Dale's-terrace, suffered from the pestilence, three of the cases ending mortally. The situation is open, clean, naturally healthful, and elevated 120 feet above high-water mark; but seven or eight yards from the front of the house was a small, stagnant, and offensive pond. Six members of another family, of respectable station, of whom four or five died, resided in a house, the atmosphere of which was vitiated by the exhalations from a foul, neglected sewer. Other cases of similar character might be adduced did time allow, but these will suffice as illustrations of the manner in which the pestilence, for the most part avoiding healthier situations, appears to have selected insalubrious localities and dwellings for its onslaught.

Two hundred and seventy-nine children, rendered orphans, became chargeable on the poor's rate in consequence of the epidemic, and one hundred and six persons lost either father or mother from the pestilence.* The immediate expense incurred by the Tynemouth union amounted to £1500. It has been calculated, that the contingent expenses of the visitation, for the maintenance of orphans and widows rendered such by this fearful visitation, would in four years amount to £13,114, thus making the gross public expenditure for a visitation of four months' continuance but little under £15,000. One half of this sum, or £7500, must have devolved upon the borough; a sum sufficiently large, if judiciously expended, to have placed the whole of the districts most severely visited in a sound sanitary state. This calculation is of course exclusive of the very serious loss sustained by the community from the deaths of many persons in more affluent circumstances, some of whom, being in the prime of life, were actively and usefully engaged in lucrative employments, whilst others, up to the time of their death, had been only sources of expenditure from the general fund, to which their early age had prevented them contributing. If it be true, as the foregoing facts tend to shew, that cholera requires certain localising causes for its more malignant development, how sad is the picture thus, although very imperfectly, brought before you. Perhaps under a better system, the majority of the lives thus sacrificed might have been preserved, as well as an amount of distress, grief, and wretchedness, which it is impossible to calculate, prevented to the survivors.

The Public Health Act was applied to the borough of

---

* These and many other facts are included in the paper which, perhaps, properly speaking, ought not to have found a place in a communication of this nature. Perhaps their interest under a sanitary aspect may be considered a sufficient reason to warrant this departure from the usual plan.

Tynemouth on the petition of the Town Council in the summer of 1851. The provisions of the act relative to the registration and regulation of common lodging houses and slaughter houses, and the construction of new streets and houses, were immediately put in force. Care was taken to prevent the erection of houses without proper conveniences and provision for ventilation; no ash-pits were allowed to be made against the main walls of dwelling houses, or without proper doors and covers; wherever sewers existed drains from the houses were insisted on, and all persons laying out new streets were compelled to have back entrances to the houses, and to provide for the construction of drains from the backs of houses, instead of carrying them underneath the basement story, as was previously usual.

In the autumn of 1852, when the reappearance of cholera in this country was considered probable, an active inspection of the town was instituted by the Public Health Act Committee: the bye courts and lanes were thoroughly cleansed, the gully grates trapped; the foul open ditch behind the North-street was cleansed and filled in, and many other local nuisances throughout the borough were removed. On the report of the first death from cholera in Newcastle in 1853, the like measures were again resorted to. The courts, lanes, and common lodging-houses were inspected by the health committee, aided by other members of the town council. Every common lodging-house in the town was peremptorily ordered to be lime-washed and cleansed within forty-eight hours, an order which was strictly obeyed. A large staff of carts and men were at once employed to cleanse all the courts, lanes, and back passages in the town, which, after the rough dirt was removed, were sluiced with water thrown into them by a powerful fire engine afloat on the river. All the courts and smaller streets, after being thus perfectly cleansed, were lime-washed. Depôts of quick-lime for the use of the poor were placed in convenient places throughout the borough, at the expense of the Board of Health: and to induce them to make free use of it, the local authorities personally visited the inhabitants of the localities in which cholera had formerly prevailed. Ruinous channels were repaired, and where the gullies were imperfectly trapped this was rectified, and chloride of lime, of which a ton was speedily consumed, was profusely used for the purpose of disinfecting them. In the course of fourteen days the town was brought into as good a sanitary state as possible under existing circumstances, 1500 cart-loads of manure having been removed in that short period from the vicinity of human habitations. The entire expense incurred by these operations amounted to £230, which was afterwards reduced to less than £200 by the sale of the manure. In addition to these energetic measures, a communication was opened with the Board of Guardians: the writer of this paper, at that time chairman of the Public Health Act Committee, being deputed to seek an interview with that body for the purpose of suggesting a co-operation between the two authorities, a recommendation most cordially acceded to by the Board of Guardians.

It is almost unnecessary to state, that Newcastle and Gateshead sustained during the autumn of 1853 the most devastating visitation of cholera that has hitherto occurred in this country. In this the whole of the surrounding districts more or less participated. At Howdon, a village within a stone's throw of the borough of Tynemouth, from which it is separated only by a narrow brook, the pestilence was rife. It was likewise prevalent in the adjoining parishes of Wallsend, Long Benton, Earsdon, and at the seaport of Blyth. Twelve deaths only occurred in the parish of Tynemouth: four of them being in a portion of Howdon, on the Tynemouth side of the streamlet. Of the remaining cases four were distinctly traceable to an imported origin; leaving thus, at the outside, only four indigenous ones. The interest in this remarkable immunity of Tynemouth is very much increased by the circumstance, that diarrhœa was exceedingly prevalent throughout the borough during the existence of cholera in Newcastle and Gateshead. From this it may, perhaps, not unfairly be inferred, that the epidemic influence was present in Tynemouth also, but that some circumstances necessary to its malignant development were absent. Judging from the facts detailed in the former portion of this paper, regarding the earlier visitations, it may with considerable probability, be inferred that the removal of so many sources of atmospheric impurity, just before the anticipated outbreak, had removed some local conditions favourable, perhaps necessary, to the development of the pestilence in its more malignant form. During the autumn of 1853, many thousands of the inhabitants of Newcastle and Gateshead, who had fled from the pestilence, took up their residence in Tynemouth. Many of them had just left the house of death or sickness, and others continued to pass and repass daily between the two towns, either to visit relatives or for business purposes: yet by none of these were the germs of the pestilence conveyed to the inhabitants of their new abode.

Certain conclusions, which appear to be fairly deducible from the foregoing history, will perhaps not unsuitably close a paper, which has already far exceeded the limits permitted by the rules of the society.

1. That cholera, neither in 1831 nor in 1849, was imported into Tynemouth by direct human intercourse, or from patient to patient.

2. That if cholera be communicable from person to person, such mode of its propagation is of insignificant importance.

3. That the propagation of cholera by means of the water, either in 1831-2 or 1849, was impossible, the reservoirs, ponds, and other sources of the Tynemouth water supply being neither polluted by sewage liquid, nor so situated as by any possibility to have become impregnated with the germs of the disease, unless these were absorbed from the atmosphere.

4. That the pestilence having almost invariably fixed its seat in the dirtiest and worst drained portions of the town, and especially in situations the atmosphere of which was more or less vitiated by decaying organic substances combined with moisture, these appear to have acted as the localising causes of cholera.

5. That poverty alone had little influence in predisposing persons to cholera, for that many of the victims were, if not wealthy, at least in comfortable circumstances.

6. That the mere overcrowding of houses, and the defective construction of streets as regards ventilation, appear only to have aggravated the disease, so far as they increased the above named localising causes.

7. That mere elevation or lowness of site, apart from the existence of foul sewers or other local causes, had no influence over the extent or severity of the disease. North-street, with an elevation varying from 60 to 70 feet, and Reed-street, with an elevation of 110 feet, in both of which the pestilence shewed itself in its worst form, having suffered much more severely than Duke-street, Clive-street, Liddle-street, and Bell-street, whose average elevation is under 25 feet above the tidal datum. Percy Main, in which the disease prevailed extensively in both epidemics, is elevated and open; and at Billy Mill, a hamlet containing scarcely a dozen cottages, and situated on the highest eminence in the parish, there were also cases on both occasions.

8. That when cholera is impending, it may either be averted, or at least the visitation be much mitigated, by active measures of a precautionary character ; and that probably sound sanitary arrangements, which provided for the complete and rapid removal of all the excretæ and other exuviæ of towns, would so improve their condition as to remove the local sources of atmospheric impurity, which appear to favour the development of cholera in a pestilential form.

9. The correctness of several of the foregoing conclusions appears to be confirmed by the singular immunity from the pestilence enjoyed by the troops, and, although less perfectly, by the inhabitants of the village of Tynemouth in 1849, and by the entire borough in 1853 ; as well as by a converse series of facts, which shew the tendency of cholera to return upon its former footsteps, and to reappear at a subsequent visitation not only in the same localities, but even in the same houses and rooms which it formerly visited, provided no essential improvement has in the meantime been made in their condition.

